# Dengue Symptoms Significance in Anti-Dengue Drug Development: Road Less Travelled

**DOI:** 10.6026/97320630013131

**Published:** 2017-05-31

**Authors:** Anubrata Paul, Arpana Vibhuti

**Affiliations:** 1 SRM University, Delhi-NCR, Sonepat, Haryana, Centre for Drug Design Discovery & Development (C-4D), PR Institute of Medical Science & Research, Delhi-NCR, Sonepat, New Delhi, India

**Keywords:** Dengue, Haryana, North India, Platelet Count, Dengue Symptoms, IgM-IgG Test, NS1 Test

## Abstract

Dengue outbreak has affected rural areas of Delhi-NCR, Haryana widely but it lacks in surveillance. High cases of dengue symptoms were reported in these regions whereas dengue symptoms have been a neglected issue in the anti-dengue drug development. Therefore, this study aims to analyze the status of the dengue infection, a rural issue of Delhi-NCR, Haryana and to identify the significance of dengue symptoms in anti-dengue drug development. The study was conducted when there is high chance of dengue infection i.e. from August 2015 to October 2015 at OPD Unit of PR Institute of Medical Science & Research, Delhi-NCR, Sonepat. It includes 158 patients from 24 rural areas of Haryana comprising both males and females from different age groups. Out of 20% cases, 6% were IgG-Positive, 9% were IgMPositive and 88% were NS1-Positive and rest 80% was normal. It includes 44% cases of thrombocytopenia. Badkhalsa village (28%), age group 18-24 (34%) and males (63%) reported cases of high infection. It was found that people with fewer platelet counts (Rai village) were not suffering from dengue whereas people with more platelet count reported dengue infection (Badkhalsa village). Interpretation & Conclusion: This study focuses on new research directions by highlighting the dengue symptoms importance in anti-dengue drug development also it is a first attempt to investigate the status of dengue, a rural issue of Delhi-NCR, Haryana and suggests that health authorities and people living in these regions should take initiatives for better health.

## Background

Dengue, a mosquito-borne viral infection caused by four serotypes of dengue virus, transmitted by Aedes aegypti has become a global concern. More than 70% of people are at risk from Asia-Pacific region [1]. The dengue outbreak has also affected a large area of India [2]. However, cases from North India are less researched excluding Delhi. National Capital Region (NCR) of India includes more than a dozen districts of Haryana. Haryana is a North Indian state surrounding Delhi. The National capital region under Haryana covers 55 villages (approximately) alongside National Highway-1. However, the surveillance from these regions is limited and has only a handful of studies. Dengue has been a major concern in these rural areas and due to unavailability of dengue drug and therapy; the frequency of cases and death from these regions are continuously multiplying.

The common dengue symptoms are sudden high fever (103-106°F), severe headache, joint pain, muscle pain, pain behind eyes, rashes and abdominal pain [3]. In 1953, Philippines confirmed the first outbreak of dengue fever [4]. In addition, these dengue symptoms are the areas of study which have been given less significance than platelets count or serotypes of dengue virus in terms of diagnosis of the disease and in the discovery of an effective anti-dengue drug and therapy [5]. Therefore, the objective of the present study is to analyze the status of the dengue infection in rural areas of Delhi- NCR, Haryana and to discover the significance of dengue symptoms in anti-dengue drug development.

Dengue fever testing is useful in determining the infection in a person who is identified with symptoms or is having recent potential exposure to dengue [6][7]. Predominantly, detection of dengue-specific IgM/IgG-class antibodies remains the most commonly utilized diagnostic method [8]. Testing of acute and convalescent sera perhaps is necessary to make the diagnosis as seroconversion occurs approximately 3 to 7 days after exposure [9]. In addition to serologic testing [10], identification of early dengue infection can be made by detection of dengue virus NS1 antigen [11]. NS1 antigenemia is detectable within 24 hours of infection and up to 9 days following symptom onset. The dengue NS1 antigen can be detected using dengue virus NS1 antigen and serum by enzyme immunoassay [12].

## Methodology

The study was conducted from thetime period of August 2015 to October 2015 at PR Institute of Medical Science & Research, Biotechnology Department of SRM University, Delhi-NCR, Sonepat. Blood samples were collected from all the dengue symptoms suspected patients for the diagnosis of dengue infection.

### Antibody Tests

To diagnose infection which is a recent or a current infection, mainly these tests are used. In response to dengue infection, the two different classes of antibodies which are produced by the body are detected by these tests (IgG and IgM). The presence of IgG-class antibodies to dengue virus is consistent with exposure to this virus sometime in the past and by 3 weeks following exposure; nearly all immunocompetent individuals should have developed IgG antibodies to DENV. On the other hand, the presence of IgM-class antibodies to DENV is consistent with acute-phase infection. Diagnosis of dengue may involve a combination of these antibodies tests, concerning the reason that over the course of the illness body's immune system produces varying levels of antibodies. Firstly, antibodies produced are IgM and if tests are performed after exposure that is 7-10 days then these tests prove to be most effective. For few weeks, blood level rises and then gradually decreases. A few months later, the IgM antibodies fall below the detectable levels. Conversely, in response to an infection, IgG antibodies are produced more slowly. The level rises with infection; it then stabilizes and later persists for along-term. Individuals exposed to the virus prior maintain IgG antibodies level in the blood which can alter or change the diagnostic results interpretation [13].

### NS1 Test

NS1 antigen test medical use is to diagnose the early infections, which are effective if detection is on the 1st day. In the primary dengue infection and on the beginning of the clinical symptoms NS1 presence is detectable in the serum of infected persons and thus, a strong humoral response is produced. Also, NS1 is detectable before IgM antibodies appear. By testing the serum samples acute dengue infection can be confirmed which were collected on the day of admission of patients with the early dengue NS1 capture ELISA [14].

### Statistical Method

IBM SPSS (version 20) has been used for statistical analysis. Firstly, descriptive statistics has been used followed by Chi-square and Cross-tabulationfor analysis of the data.

## Results

### Antibody Testing

The presence of antibody type (IgG or IgM) helps in interpreting antibody tests as positive or negative or an antibody titer. If in an initial blood sample positive IgM and IgG tests are detected for dengue antibodies then more probably it means that within the recent weeks, the person has been infected with dengue virus. Considering another case in which IgG is positive but IgM is negative or low, then it can be concluded that sometime in the past person had an infection. If there is four-fold or greater increase in the dengue IgG antibody titer that is the titer of 1:4 to a titer of 1:64, between samples (one- initial sample and another - sample taken 2 to 4 weeks later), then probably that person had a recent dengue infection. Negative tests for IgM and/or IgG antibodies indicate that individual has no dengue infection and symptoms can be due to another cause or in an another case, it can be interpreted that antibody level is too low to be measurable.

### NS1 Testing

The presence of dengue NS1 antigen is consistent with acute-phase infection with dengue virus. Positive test for DENV may mean that NS1 antigen is typically detectable within 1 to 2 days following infection and up to 9 days following symptom onset. NS1 antigen may also be detectable during secondary dengue virus infection, but for a shorter duration of time (1-4 days following symptom onset). Negative test for DENV may mean that the absence of dengue NS1 antigen is consistent with the lack of acute-phase infection. The NS1 antigen may be negative if the specimen is collected immediately following dengue virus infection (<24-48 hours) and is rarely detectable following 9 to 10 days of symptoms.

### Statistical Analysis

**Figure 1:**
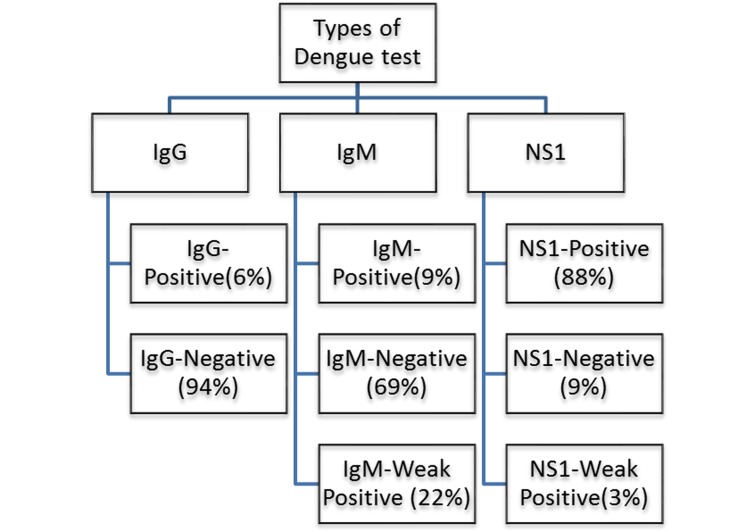
Summary of Dengue test results is illustrated using a block diagram

During the study period, a total of 158 patients reporting dengue symptoms were admitted comprising 56% males and 44% females. The majority of patients were from the age group 18-24 (30%) followed by 25-39 (26%). Out of 24 rural areas, the highest cases were from Badkhalsa village (18%), Jakholi village (11%) and BheraBankipur village (10%). The demographic details of the patients are given in Table 1. Table 2 shows the dengue symptoms which have been classified into six major categories for our analysis. Headache (35%), fever (32%) and joint pain (21%) were the most commonly reported symptoms among these patients. Table 3 summarizes the 32 (20%) dengue cases; while 80% were reported normal. Table 4 represents platelet count and age wise (P<0.05) distribution of dengue cases followed by chi square test in Table 5. Dengue test was found positive -IgG (6%), IgM (9%) and NS1 (88%) while weak positive results were found -IgM (22%) and NS1 (3%) as shown in Figure 1. Positive cases were found more in males (63%) than females (38%).

Out of these 32 cases, 14 (44%) cases were of thrombocytopenia (having platelets less than 1,50,000) as shown in Table 6 while platelets less than 50,000 were 7%, platelets count between 51,000- 1,00,000 were 21%, 1,01,000-1,50,000 were 71%, respectively. These cases were from the age group 25-39 (43%) and from region Rai (29%). In IgG-Positive, patients having platelets count 251000- 300000 were suffering from symptoms such as fever, in IgMPositive patients having platelets count 2,01,000-2,50,000 and 2,51,000-3,00,000 were having symptoms such as joint pain, fever and muscle pain and in NS-Positive patients having platelets 1,51,000-2,00,000 and 2,01,000-2,50,000 were suffering from headache, joint pain and fever; while patients with platelet counts 2,51,000-3,00,000 were diagnosed with symptoms such as muscle pain and fever.

## Discussion

The outbreak of dengue can be vigilantly measured by the number of dengue cases and death, and its significance can be considered by the urgent need for the development of the anti-dengue drug. However, without proper surveillance and understanding of the root cause of the disease, there will be a certain delay in the process of anti-dengue drug development. The present study is the initial step towards contributing awareness of dengue in the rural areas of Delhi-NCR, Haryana. However, the study was conducted for three months but still it has shown high cases of dengue. Health authorities and people living in these regions should take initiatives for better health and prevention from dengue. On the contrary, from the analysis it can be concluded that the region with high dengue cases (Badkhalsa) was not the same region with high cases of thrombocytopenia instead it was a village named “Rai”. Thus, the analysis revealed that the regions with high cases of dengue, both having dengue symptoms and dengue positive test were not the same region having platelets count less than 1,50,000 i.e. below the normal count. The role of anti dengue drug has been less studied in the treatment of dengue symptoms such as dengue fever [5].Therefore, we conclude from our analysis that the dengue symptoms which were identified from these cases such as high fever, joint pain, and headache are more crucial to be researched on and future studies are encouraged on finding an effective treatment for these symptoms.

### Limitations

There is a chance or limitation that if the antibody test for dengue fever comes to be positive then there is a possibility that the person has been infected with West Nile virus, an arbovirus. In that case, individual test results, their medical history, and latest travel history should be considered by health practitioners while making a diagnosis.

Test results should be used in conjunction with clinical evaluation, including exposure history and clinical presentation. False-positive results, particularly with the dengue virus IgG antibody test, may occur in persons infected with other Flaviviruses, including West Nile virus and St. Louis encephalitis virus. Obtaining a detailed exposure history and further laboratory testing may be necessary to determine the infecting virus. Positive test results may not be valid in persons who have received blood transfusions or other blood products within the last several months. The significance of a negative result in an immunosuppressed patient is unclear. Though uncommon, false-positive NS1 results may occur in individuals with active infection due to other flaviviruses, including West Nile virus and yellow fever virus. Negative NS1 antigen results may occur if the specimen was collected >7 days following symptom onset. In such cases, Serologic testing is recommended to detect the presence of IgM and IgG antibodies to dengue virus. Comparatively, if the diagnosis of dengue virus infection is conducted within the first five days of illness, NS1 test is an effective method than antibody tests. In addition, the early diagnosis and management can cause a reduction in the morbidity and mortality of dengue fever. However, there will be an increase in the sensitivity of diagnosis from sixth day onwards, if used, a combination of antibody tests and NS1 test.

## Conclusion

Alarming results in the region of Delhi-NCR focuses on new research directions by highlighting the dengue symptoms importance in anti-dengue drug development. The study contributes a bit to the body of knowledge by initiating an attempt to investigate the status of dengue in Delhi-NCR, Haryana. Concluding, health authorities and people living in these regions should take initiatives for better health.

## Conflict of Interest

We declare that we have no conflict of interest

## Figures and Tables

**Table 1:** Demographic details of the patients (n=158)

Parameters	Characteristics	Frequency	Percentage
Gender	Females	69	44
	Males	89	56
Age	0-12	20	13
	13-17	14	9
	18-24	47	30
	25-39	41	26
	40-59	24	15
	60 and above	12	8
Region	Asawarpur	1	1
	Aterna	2	1
	Badkhalsa	28	18
	Baduali	1	1
	BheraBankipur	16	10
	Biswamil	5	3
	GaddiKheri	1	1
	Jagdishpur	5	3
	Jakholi	17	11
	Jathari	11	7
	Khathar	2	1
	Khewra	2	1
	Kundli	7	4
	Makirpur	2	1
	Nandnor	1	1
	Nangal	1	1
	Narela	3	2
	Papnera	1	1
	Patla	8	5
	Rai	21	13
	Rasolpur	1	1
	Seuli	11	7
	Sonepat	4	3
	National Capital Region	7	4

**Table 2:** Dengue symptoms reported (n=158)

Symptoms	Frequency	Percentage
Headache	55	35
Joint Pain	33	21
Muscle Pain	11	7
Rash	5	3
Abdominal Pain	3	2
Fever	51	32

**Table 3:** Demographic details of Patients suffering from Dengue (n = 32)

Parameters	Characteristics	Frequency	Percentage
Gender	Females	12	38
	Males	20	63
Age	0-12	8	25
	13-17	2	6
	18-24	11	34
	25-39	7	22
	40-59	3	9
	60 and above	1	3
Region	Badkhalsa	9	28
	Biswamil	1	3
	Jakholi	1	3
	Jathari	3	9
	Khathar	1	3
	Kundli	2	6
	Narela	3	9
	Patla	1	3
	Rai	6	19
	Seuli	4	13
	National Capital Region	1	3

**Table 4:** Platelets count and age wise distribution of dengue cases (n=32)

Platelets Count	Age Groups						Total
0-12	13-17	18-24	25-39	40-59	60 and Above	
Less than 50,000	0	0	0	0	1	0	1
	0%	0%	0%	0%	33%	0%	3%
51,000-1,00,000	0	0	1	2	0	0	3
	0%	0%	9%	29%	0%	0%	9%
1,01,000-1,50,000	0	0	4	4	2	0	10
	0%	0%	36%	57%	67%	0%	31%
1,51,000-2,00,000	2	0	3	1	0	1	7
	25%	0%	27%	14%	0%	100%	22%
2,01,000-2,50,000	1	2	2	0	0	0	5
	13%	100%	18%	0%	0%	0%	16%
2,51,000-3,00,000	5	0	1	0	0	0	6
	63%	0%	9%	0%	0%	0%	19%

**Table 5:** Chi-Square Tests

Chi-Square Tests			
	Value	df	Asymp. Sig. (2-sided)
Pearson Chi-Square	46.296a	25	0.006
Likelihood Ratio	40.423	25	0.026
Linear-by-Linear Association	14.848	1	0
N of Valid Cases	32		
a. 36 cells (100.0%) have expected count less than 5. The minimum expected count is .03.			

**Table 6:** Demographic details of thrombocytopenia cases (n=14)

Parameters	Characteristics	Frequency	Percentage
Gender	Females	5	36
	Males	9	64
Age	18-24	5	36
	25-39	6	43
	40-59	3	21
Region	Badkhalsa	3	21
	Jathari	2	14
	Khathar	1	7
	Narela	1	7
	Patla	1	7
	Rai	4	29
	Seuli	2	14
